# Development and initial psychometric assessment of the race-related attitudes and multiculturalism scale in Australia

**DOI:** 10.1371/journal.pone.0230724

**Published:** 2020-04-01

**Authors:** Dandara Gabriela Haag, Pedro Henrique Ribeiro Santiago, Davi Manzini Macedo, João Luiz Bastos, Yin Paradies, Lisa Jamieson

**Affiliations:** 1 Australian Research Centre for Population Oral Health (ARCPOH), Adelaide Dental School, University of Adelaide, Adelaide, Australia; 2 Department of Public Health, Federal University of Santa Catarina, Florianópolis, Brazil; 3 Alfred Deakin Institute for Citizenship and Globalisation, Deakin University, Geelong, Australia; University of Lleida, SPAIN

## Abstract

**Aim:**

The present study aims to develop the Race-related Attitudes and Multiculturalism Scale (RRAMS), as well as to perform an initial psychometric assessment of this instrument in a national sample of Australian adults.

**Methods:**

The sample comprised 2,714 Australian adults who took part in the 2013 National Dental Telephone Interview Survey (NDTIS), which includes a telephone-based interview and a follow-up postal questionnaire. We used Exploratory Factor Analysis (EFA) to evaluate the RRAMS’ factorial structure (n = 271) and then proceeded with Confirmatory Factor Analysis (CFA) to confirm the proposed structure in an independent sample (n = 2,443). Measurement invariance was evaluated according to sex, age and educational attainment. Construct validity was assessed through known-groups comparisons. Internal consistency was assessed with McDonald’s Ω_H_ and ordinal *α*. Multiple imputation by chained equations was adopted to handle missing data.

**Results:**

EFA indicated that, after excluding 4 out of the 12 items, a two-factor structure provided a good fit to the data. This configural structure was then confirmed in an independent sample by means of CFA (χ^2^(19) = 341.070, p<0.001, CFI = 0.974, RMSEA = 0.083; 90% CI [0.076, 0.091]). Measurement invariance analyses suggested that the RRAMS items can be used to compare men/women, respondents with/without tertiary education and young/older participants. The “Anglo-centric/Assimilationist attitudes” (Ω_H_ = 0.83, α_ORDINAL_ = 0.85) and “Inclusive/Pluralistic attitudes” subscales (Ω_H_ = 0.77, α_ORDINAL_ = 0.79) showed adequate reliability. Men and participants with low education had higher Anglo-centric/assimilationist attitudes and lower inclusive/pluralistic attitudes, suggesting construct validity.

**Conclusions:**

The RRAMS appears to be a valid and reliable measure to evaluate multiculturalist attitudes in the Australian context. The instrument may be useful in the assessment and monitoring of interventions aiming to promote multiculturalist inclusive attitudes and to increase social cohesion in Australia.

## Introduction

Racism emerges whenever social and individual values, norms and practices of a given group are considered superior to others’. Racism occurs with the particular aim of creating, maintaining or reinforcing power imbalances, as well as the corresponding inequalities in opportunities and resources along racial lines [1]. Similar to most contemporary societies, Australia is characterized by co-existing expressions of cultural diversity on the one hand, and negative impacts of racism on social cohesion on the other [1]. In Australia, the mental health costs directly attributable to racism have been estimated at 235,452 disability-adjusted life years lost, which is equivalent to an average $37.9 billion in productivity loss per annum, or 3% of the Australian annual Gross Domestic Product (GDP) over 2001–2011 [2]. Such a strong relationship is an indication that racism may erode the very social fabric of the Australian society by producing mental disorders and suffering, which unevenly impacts upon racially marginalized groups.

Social conceptions that shape intergroup relations form the common ground upon which intergroup attitudes and discriminatory behaviour take place [3]. From an empirical viewpoint, findings suggest that racist attitudes are associated with racist behaviours and racial-ethnic minorities’ experiences of discrimination [4]. Positive attitudes towards diversity, however, are negatively associated with discriminatory behaviour [5]. In this study, we propose to explore attitudes in relation to multiculturalism, a construct of special relevance to the social, economic and political fabric of contemporary Australia [6]. We focus on multiculturalism as an ideology of acknowledging and celebrating ethnic and cultural differences, in which the need for preserving cultural identities is recognized [7]. It reflects a “sensibility and [a] disposition towards cultural differences among large sections of the population”[8]. Data from the 2016 Australian Census revealed that one in three Australians were born overseas, and a similar proportion of individuals speak a language other than English at home. Nevertheless, assimilationist attitudes—expectations of conformation to the dominant culture—often prevail, as opposed to multiculturalist perspectives that accept and praise racial and ethnic-cultural diversity [9]. Understanding attitudes to multiculturalism can contribute to unveil the dynamics of racism and discrimination against minorities in the country, fostering public debate and policy formulation aimed to promote positive intergroup relations [10].

Research on ethnic-racial intergroup attitudes draws from theories on ideological attitudes that explain group-based dominance and social cohesion [11–13]. Social Dominance Orientation (SDO), for example, reflects the degree to which respondents believe that hierarchy-based dominance between social groups is natural [14]. Discrimination against minorities, therefore, can be explained by the degree of endorsement of the notion that group-based hierarchies are natural and inevitable [14]. Endorsement of group-based dominance and out-group prejudice tends to increase among those who highly identify with the dominant group, as they represent a mechanism of maintaining the in-group *status quo* [12].

Research on ethnic-racial intergroup relations in contemporary societies has also explored the Right-wing Authoritarianism (RWA) concept [15–17]. RWA is characterized by the endorsement of social conservative values, morality, collective security, group-based social cohesion, and strict obedience to social authorities [15, 17]. Those who endorse RWA values can be more sensitive to threats to social stability, being prone to conservative values as to increase their perception of control and collective security [18]. Perception of threat has been shown to mediate the association between group identification and attitudes towards multiculturalism [11]. Those that consider immigrants or ethnic-racial minorities as a threat to the control of resources or maintenance of the dominant social values tend to endorse more conservative/assimilationist attitudes towards multiculturalism [11, 19].

Sustaining dominant group *status quo* can also be achieved by not acknowledging ethnic-racial inequalities in the population. The so-called colour-blind racial ideology denies the existence of racism and justifies racial inequalities as a result of personal decisions, meritocratic achievements, and market forces [20, 21]. By denying racist practices and racial inequalities, it provides the discursive tools to downplay policy proposals aimed at promoting racial justice and therefore maintains the power imbalance between ethnic-racial groups [20]. Following this perspective, public denial of racism has been pointed as an obstacle to a deeper commitment to multiculturalism in Australia [13, 22]. Although the existence of racism is acknowledged, most Australians fail to recognise the existence of Anglo-privilege, a necessary step in reducing the imbalance in resource distribution and political representation among ethnic-racial groups [13].

Taken together, the results mentioned above point to the centrality of properly assessing the different facets of intergroup attitudes towards multiculturalism as to inform public debate and contribute to prevent and counteract discrimination. It is important to note that the majority of the available scales used to assess race-related attitudes have been developed and psychometrically examined among U.S. populations [7]. These tools may not be relevant or provide valid/reliable estimates of race-related attitudes in non-US contexts, though, given the considerable contextual dependency of racism. Historiographic and sociological accounts of racial dynamics usually emphasize Australian specificities in terms of colonization, past and contemporary immigration policies, and patterns of cultural diversity as key aspects.

Australia is a settler society that started with a policy of Anglo-celtic migration only. This was later expanded to include migrants from other European-backgrounds (e.g., Greeks, Italians), having only in the 1980s opened its borders to migrants of Asian and Middle-Eastern descent. These and other specificities (e.g., limited involvement in slave trade) cast serious doubts on the idea of simply adapting tools developed in a range of different countries to the Australian context. Just like other multiculturalist societies, including Canada and New Zealand, multiculturalism was debated at a national level as a state-policy in the 1970s. Backlashes from conservative sectors, nonetheless, contributed to prioritise an assimilationist perspective on the implementation of multiculturalism values in society. Australia has also historically dispossessed and oppressed the native Aboriginal Australians since British colonization with ongoing effects until present [23]. Our study does not focus on colonisation and racism faced by Aboriginal Australians as the unique features of these experiences can be diminished when considered under the umbrella of multiculturalism [24].

To the best of our knowledge, two measurement instruments that provide information on racial, ethnic, and cultural acceptance (i.e. race-related and multiculturalist attitudes) have been previously developed and assessed in Australia [7, 25]. While the first has focused on intercultural understanding among teachers and students in schools [25], psychometric evaluation of the second was carried out in relatively young and convenience samples of primary and secondary school students (all younger than 15 years-old residing in Victoria) and community members (mean age of 23 years-old with 70% residing mainly in Victoria), which limits their applicability at a national level and among older age groups. Therefore, neither an integrated picture of attitudes towards multiculturalism across the country has yet been delineated, nor a range of strategies to advance racial equity based on this knowledge have been proposed.

The present study proposes the Race-related Attitudes and Multiculturalism Scale (RRAMS) as a measure of attitudes towards multiculturalism. The items were formulated to reflect social ideologies and collective beliefs that potentially influence ethnic-racial intergroup attitudes. The aim of this study was to verify its applicability to the Australian context by assessing the extent to which the RRAMS provides a valid and reliable measurement of multiculturalist attitudes in a sample of Australian adults across all states and territories. In particular, the internal validity of the RRAMS was assessed in terms of its configural structure (i.e., the number of underlying factors), metric properties—the magnitude of factor loadings—, as well as measurement invariance (i.e., whether it allowed meaningful comparisons across sociodemographic characteristics). External validity of the RRAMS was then assessed in term of its construct validity.

## Methods

### Study design and participants

This was an Australian population-based study, with data obtained from the 2013 National Dental Telephone Interview Survey (NDTIS), which includes a telephone-based interview and a follow-up postal questionnaire. The NDTIS has been carried out periodically by the University of Adelaide since 1994, and comprises a large national sample of Australian residents aged 5 years and over. The NDTIS is a random sample survey that collects information on the dental health and use of dental services of Australians in all states and territories. The survey also collects data on social determinants of oral health and wellbeing, which include detailed information on sociodemographic factors, such as household income, education, country of birth, remoteness of location and main language spoken at home. For the 2013 survey, an overlapping dual sampling frame design was adopted. The first sampling frame was created from the electronic product ‘Australia on Disc 2012 Residential;’ an annually updated electronic listing of people/households listed in the White Pages across Australia. Both landline and mobile telephone numbers were provided on records where applicable.

A stratified two-stage sampling design was used to select a sample of people from this sampling frame. Records listed on the frame were stratified by state/territory and region, where region was defined as Capital City/Rest of State. A systematic sample of records was selected from each stratum using specified sampling fractions [26]. To include households that were not listed in the White Pages, a second sampling frame comprising 20,000 randomly generated mobile telephone numbers was used. This sampling frame was supplied by *Sampleworx* and the mobile telephone numbers were created by appending randomly generated suffix numbers to all known Australian mobile prefix numbers. As the mobile numbers did not contain address information, the sampling frame could not be stratified by geographic region. A random sample of mobile numbers was selected from the frame and contacted to establish the main user of the mobile phone. This person was asked to participate in the telephone interview, provided that they were aged 18 years or over. All participant provided verbal consent to participate in the survey and datasets were de-identified to ensure anonymity [26].

Following the completion of the telephone interview survey, participants were invited to respond to the postal questionnaire component. Those who agreed were sent a covering letter with the questionnaire and reply-paid envelope enclosed. A reminder postcard was sent two weeks later, with, if necessary, two additional follow-up letters/questionnaires sent subsequent to the postcard. A total of 6,340 Australian adults aged 18+ years took part in the 2013 NDTIS, with 2,935 (46.3%) completing the follow-up postal questionnaire. Sample characteristics are displayed in Table 1. Two thirds of the sample were 45 to 98 years old and had Technical and Further Education (TAFE) or went to university. Women corresponded to 60.3% of the sample. The majority of participants were born in Australia (76.7%), 12.8% were originally from Europe and 10.5% from the other continents (Asia, Africa and the Americas).

**Table 1:** Characteristics of study participants (n = 2,714).

	Total sample		EFA** subsample		CFA*** subsample	
Sample characteristics	n	%	n	%	n	%
**Age**						
18 to 45 years	809	29.8	101	37.2	708	29.0
46 to 98 years	1818	67.0	162	59.8	1656	67.8
Missing	87	3.2	8	3.0	79	3.2
**Sex**						
Female	1637	60.3	176	64.9	1461	59.8
Male	990	36.5	87	32.1	903	37.0
Missing	87	3.2	8	3.0	79	3.2
**Education**						
High school or less	548	20.2	60	22.1	1876	20.0
TAFE* or university	2079	76.6	203	74.9	488	76.8
Missing	87	3.2	8	3.0	79	3.2
**Country of birth**						
Australia	2079	76.6	209	77.1	1870	76.5
Rest of Oceania	72	2.6	6	2.2	66	2.7
Europe	347	12.8	36	13.3	311	12.7
Africa & Middle East	43	1.6	1	0.4	42	1.7
Asia	56	2.1	5	1.8	51	2.1
Americas	30	1.1	6	2.2	24	1.1
Missing	87	3.2	8	3.0	79	3.2

*TAFE, Technical and Further Education (trade school/college).

**EFA, Exploratory Factor Analysis. This refers to respondents whose data were analyzed with EFA in Phase 2 of statistical analysis.

***CFA, Confirmatory Factor Analysis. This refers to respondents whose data were analyzed with CFA in Phase 3 of statistical analysis.

### Ethical approval

Ethical approval for the study was granted by the University of Adelaide’s Human Research Ethics Committee (approval number HS-2013-036).

### Statistical analysis

Statistical analyses were conducted with R software [27] and R packages lavaan [28], and semTools [29].

#### Phase 1: Item development

The RRAMS was developed by a group of researchers with expertise on the topics of racism, multiculturalism, and race-related attitudes in Australia. To ensure content validity [30], the scale was based on large surveys carried out in the country that were co-designed by the abovementioned group of researchers. These include the 2015–16 Challenging Racism Project [31] and the 2013 survey of Victorians’ attitudes to race and cultural diversity [32]. The initial item development phase consisted in the design of items that reflect the different social ideologies that encompass multiculturalism and race-related attitudes. Discussions among the panel of experts were held until reaching consensus that the items comprehended a varied number of theoretical perspectives underpinning the construct of interest. A second group of experts—not involved in the first development phase—was then consulted for feedback purposes in relation to comprehensiveness and clarity of the items.

The final RRAMS was proposed as comprised by two subscales. The first subscale included six items reflecting theories and social ideologies in agreement with “Anglo-centric/Assimilationist attitudes.” It included items reflecting alignment with RWA (e.g., ‘We need to stop spreading dangerous ideas and stick to the way things have always been done in Australia’), agreement with SDO (‘It is okay if some racial or ethnic groups have better opportunities in life than others’), endorsement of colour-blind racial ideology (e.g., ‘We shouldn’t talk about racial or ethnic differences’), zero-sum racist thinking (e.g., ‘Racial or ethnic minority groups take away jobs from other Australians’), and endorsement of assimilationist ideology (e.g., ‘People from racial or ethnic minority groups should behave more like mainstream Australians’).

The second subscale comprised six items assessing agreement with “Inclusive/Pluralistic attitudes.” It included low compliance to RWA (e.g., ‘Some of the best people in our country are those who are challenging our government and ignoring the ‘normal’ way things are supposed to be done’), low SDO (e.g., ‘We should do what we can to create equal conditions for different racial or ethnic groups’), acknowledgment of racism (e.g., People from racial or ethnic minority groups experience discrimination in Australia), acknowledgment of white privilege (e.g., ‘Australians from an Anglo background (that is, of British descent) enjoy an advantaged position in our society’), and endorsement of multiculturalism (e.g. “People from racial or ethnic minority groups benefit Australian society”). Besides their theoretical relevance, these constructs have been found to be acceptable and appropriate for assessing population race-related attitudes in previous national studies in Australia [31,32]. Response options for each item ranged from ‘strongly disagree’ (0), ‘disagree’ (1), ‘neither agree nor disagree’ (2), and ‘agree’ (3) to ‘strongly agree’ (4).

#### Phase 2: Identification of a potential factorial structure

Since the RRAMS was conceptualized to measure agreement with both conformity to the dominant ethnoculture (“Anglo-centric/Assimilationist attitudes”) and agreement with promotion of ethnic diversity (“Inclusive/Pluralistic attitudes”), an Exploratory Factor Analysis (EFA) was initially run to *empirically* test this assumption (i.e., that a two-factor solution would underlie the set of items). The factorial solution suggested by the EFA was then confirmed by means of a Confirmatory Factor Analysis (CFA) [33] in an *independent sample* to avoid capitalization on chance [34, 35]. We randomly divided the NDTIS sample into one group for the EFA and another group for the CFA; see Table 1 for the distribution of each subsample according to sociodemographic characteristics. Considering that a sample size with at least 200 participants is sufficient for EFA under normal conditions (medium communalities and at least three items loading on each factor) [36] and CFA has higher sample requirements, 271 participants from the original survey were randomly selected for the EFA.

Factor retention relied on Scree Plot [37] criteria and Parallel Analysis (PA) [38]. In the PA, 1,000 random and resampled datasets with the same number of RRAMS items and respondents were generated. The rationale of the PA is that meaningful factors extracted in the current study should account for more variance than factors extracted from random data [36]. Factor extraction was conducted with Maximum Likelihood [39] and oblique rotation (“direct oblimin”) [40]. Items with non-salient factor loadings (.<40) were deleted. Additionally, 100 bootstrapped samples were used to generate factor loadings’ 95% confidence intervals [41].

#### Phase 3: Confirmation of the factorial structure in an independent sample

After a factorial structure was derived from the EFA, the instrument was assessed using CFA in an independent sample (n = 2,443). The estimation method was Weighted Least Squares [42], with a mean- and variance-adjusted (WLSMV) test statistic [43]. Missingness of individual item responses ranged from 0.9% to 2.2%, and this was handled with multiple imputation of 20 datasets using the fully conditional specification method [44]. We imputed information for individuals who responded to at least one item of the RRAMSs (n = 2,714). Rubin’s rules [45] were used to pool point estimates and standard errors (SE). To evaluate model fit, the scaled χ^2^ was used to test the hypothesis of *exact-fit*. Additionally, we used *approximate fit* indices, such as the scaled Comparative Fit Index (CFI) and scaled (for simplicity, the term ‘scaled’ will be omitted from now on.) Root Mean Squared Error of Approximation (RMSEA). Values of CFI ≥ 0.96 and RMSEA ≤ 0.5 indicate good model fit [46], while 0.5 < RMSEA ≤ 1.0 indicates acceptable fit [35].

Since factorial structures derived from EFA do not necessarily imply good fitting CFA models (e.g. due to cross-loadings or residual correlations) [47], in case the factorial structure had a poor fit, model re-specifications were informed by standardized residuals, Modification Indices (MI) and the Standardized Expected Parameter Change (SEPC) [48]. Completely standardized solutions were reported throughout the paper.

#### Phase 4: Analysis of measurement invariance

An initial Multigroup CFA [49] was conducted to check if the same configural structure would hold for all sex, age, and education-based groups—i.e., this was done to check whether *configural invariance* could be confirmed with the data at hand. The χ^2^, CFI and RMSEA and their previously described cut-off points were used to evaluate configural invariance. The second level of measurement invariance, *metric invariance*, was assessed to ascertain whether factor loadings were similar across the same groups. The final test, *scalar invariance*, was used to determine whether item thresholds were equal across sex, age and education. Given that scalar models are nested within metric models, and metric models are nested within configural models, metric and scalar invariance were evaluated through a Likelihood Ratio Test (LRT), namely the **Δ** χ^2^ [50]. The **Δ** χ^2^ statistic was computed in each imputed dataset and pooled according to Li, Meng [51] recommendations (i.e. D2 statistic). When the **Δ** χ^2^ was *statistically* significant, the **Δ**CFI [52] was used to evaluate the *magnitude* of the difference. Models with **Δ**CFI ≤ -.002 indicated lack of invariance [53]. Whenever measurement invariance was not achieved, tests of partial invariance were conducted [54].

#### Phase 5: Reliability

Internal consistency was calculated with McDonald’s Ω_H_ [55] and ordinal ***α*** [56]. The McDonald’s Ω_H_ has two advantages over the traditional and widely used Cronbach’s ***α***: It does not assume (1) tau-equivalence or a (2) congeneric model without correlated errors (i.e. locally independent items) [57]. Furthermore, the ordinal ***α*** is reported given that Cronbach’s ***α*** underestimates reliability in ordinal Likert scales. Adequate methods for calculating ordinal ***α*** confidence intervals are not available [58].

#### Phase 6: Item reduction analysis

In the item reduction analysis, we evaluated inter-item correlations, corrected item-total correlations (CITC) and item difficulties. Inter-item correlations indicate the extent to which all items on a scale are examining the same construct without redundancy. Thus, inter-item correlations should be moderate (i.e. items that measure the same construct but also have unique variances) and items with correlations lower than .20 were considered for deletion [59].

The next step was the evaluation of CITC. One important aspect in instrument development is achieving a good balance between a small number of items (lengthy questionnaires can induce lower response rates [60]) and adequate reliability. A recent study by Zijlmans, Tijmstra [61] showed that the CITC [62] performed better than other methods at identifying which items can be removed while maximizing reliability. Therefore, items with the lowest CITC should be the first to be considered for removal. The corrected item-*total* correlation needs to be calculated *within subscales*, since items can only be summed into a *total* score when they measure the same construct [63]. For this reason, CITCs were calculated *after* the factorial structure was established (i.e. we had no prior information about which item belonged to which subscale to calculate corrected total scores). Given the ordinal nature of the data, the inter-item correlations and CITCs were investigated with non-parametric Kendall’s τ [64].

Finally, due to the limitations of classical difficulty indices such as the p-value (i.e. proportion of correct responses given the total score) [65], we evaluated item difficulty with the LI_IRF_, the location index based on the item-response function [66]. The LI_IRF_ is calculated based on the *item locations* (β_i_), which are a well-known reparameterization of *item thresholds* (τ_i_) of adjacent *i* and *i* +1 response categories [67]. The LI_IRF_ indicates the value of the latent trait in which respondents have an *average* score of half the maximum item score. For example, in a 5-point rating scale (items ranging from 0 = Strongly Disagree to 4 = Strongly Agree), the LI_IRF_ indicates the level of inclusive/pluralistic attitudes required for participants to score *on average* 2 (2 = Neutral). In our study, the LI_IRF_ was chosen over item thresholds (τ_i_) to convey item difficulty because of two advantages: the interpretation of the LI_IRF_ is (a) easier, since it is a single index compared to four thresholds per item; and (b) more substantive, since it is based on the *latent trait* (“Anglo-centric/Assimilationist attitudes” or “Inclusive/Pluralistic attitudes”) rather than on the *latent response variables* [68]. Nonetheless, for the sake of completeness, we also reported the item thresholds (τ_i_).

#### Phase 7: Construct validity

To evaluate the RRAMS’ construct validity, we investigated known-groups validity according to sex, education and age. Known-groups validity compares the levels of the constructs in different groups (e.g. men compared to women) and should be applied when it is known, theoretically or due to previous empirical research, that these groups differ on the variable of interest. Therefore, known-groups validity can inform whether the instrument is able to *discriminate* between two groups that are *known* to be different regarding the construct (e.g. individuals with more education have more inclusive attitudes). Investigation of known-groups validity is important in many instances, such as when there is no “gold-standard” method of measurement to which the instrument can be compared [69]. That is, since there is no “gold-standard” or established (based on robust psychometric evidence) instrument to measure race-related attitudes and multiculturalism in Australia, it is not possible to define what would constitute a good measure for the RRAMS to display convergent validity with. Furthermore, in our case, there is previous evidence of groups that are known to differ according to multiculturalism and race-related attitudes. For example, as multiculturalism can be perceived as identity-threatening by dominant group members [11, 19], we expected men to have more conservative attitudes towards multiculturalism when compared to women [22, 70]. The same pattern was expected for older participants (>45 years old) when compared to younger respondents [22, 70, 71]. Participants with a university degree, in turn, were expected to be more supportive of multiculturalism than those with lower educational attainment. This hypothesis is in accordance with previous findings showing that sense of economic security (economic, personal, and cultural), higher education and younger age were associated with more positive attitudes towards multiculturalism and lesser exclusionary attitudes [22, 70, 71]. Therefore, sex, age and education were chosen as the exogenous variables for the evaluation of known-groups validity. To assess known-groups validity, latent mean differences were calculated by constraining the latent means in one of the groups (i.e. women and participants with higher education) to zero, so this group would function as a reference group. Considering that latent variances were constrained to one in the completely standardized solution, latent mean differences are interpreted as effect sizes analogous to Cohen’s [72] *d* [73]. Finally, we employed the Empirical Bayes model [74] to estimate factor scores, which were plotted using Kernel density [75] to inform not only the *average* but also the distribution of the latent trait according to groups.

## Results

### Identification of a potential factorial structure

**Fig 1:**
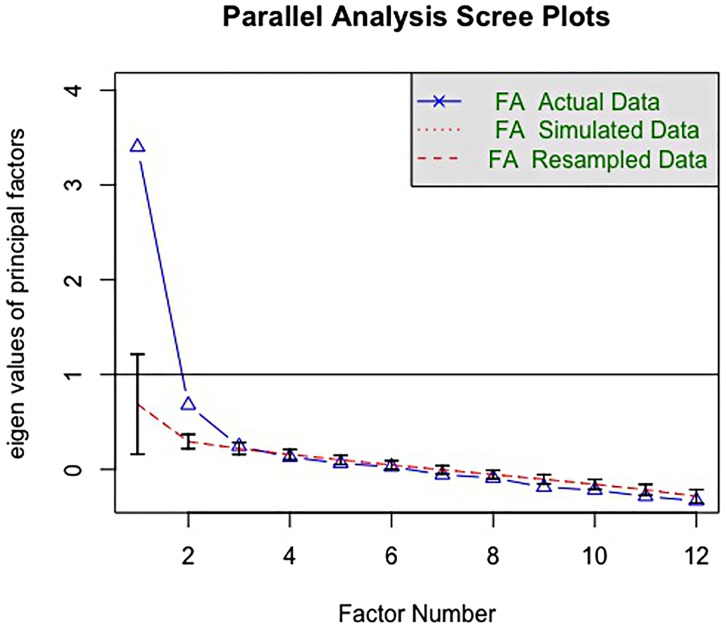
Parallel analysis and Scree Plots of the race-related attitudes and multiculturalism scale. The triangles indicate the factors’ eigenvalues extracted from the study data. The dashed lines and 95% CI indicate the factors’ eigenvalues extracted from the 1,000 simulated and resampled datasets. FA stands for factor analysis.

Investigation of the Scree Plot and PA indicated that 2 factors substantially explained more variance than factors extracted from randomly generated data (Fig 1).

It should be noted that, although the third factor accounted for more variance than the third factor extracted from the random datasets, the difference was trivial. For this reason, only two factors were retained. The next step consisted of the evaluation of factor loadings (Table 2). Results showed that Item 2 (“Some of the best people in our country are those who are challenging our government and ignoring the ‘normal’ way things are supposed to be done”), Item 3 (“It is okay if some racial or ethnic groups have better opportunities in life than others”) and Item 6 (“We shouldn’t talk about racial or ethnic differences”) did not have substantial factor loadings (>.40) and were therefore excluded. Item 5 had the smallest factor loadings (λ_2_ = 0.440 95% CI [0.220, 0.610]).

**Table 2:** Exploratory Factor Analysis: Factor loadings (λs) and bootstrapped 95% CI (n = 271).

Item	Factor 1		Factor 2	
	Estimate	95% CI	Estimate	95% CI
1. We need to stop people spreading dangerous ideas and stick to the way things have always been done in Australia.	0.59	[0.40, 0.77]	-0.10	[-0.26, 0.03]
2. Some of the best people in our country are those who are challenging our government and ignoring the ‘normal’ way things are supposed to be done.	**0.08**	**[-0.16, 0.26]**	**0.38**	**[0.15, 0.57]**
3. It is okay if some racial or ethnic groups have better opportunities in life than others.	**0.27**	**[0.00, 0.47]**	**0.10**	**[-0.16, 0.30]**
4. We should do what we can to create equal conditions for different racial or ethnic groups.	-0.12	[-0.28, 0.02]	0.57	[0.39, 0.74]
5. Australians from an Anglo background (that is, of British descent) enjoy an advantaged position in our society.	-0.03	[-0.25, 0.15]	0.44	[0.22, 0.61]
6. We shouldn’t talk about racial or ethnic differences.	**0.23**	**[-0.02, 0.44]**	**-0.06**	**[-0.28, 0.13]**
7. People from racial or ethnic minority groups benefit Australian society.	-0.06	[-0.27, 0.11]	0.47	[0.24, 0.64]
8. People from racial and ethnic minority groups experience discrimination in Australia.	0.01	[-0.16, 0.11]	0.74	[0.57, 0.88]
9. Something more should be done to reduce discrimination experienced by people from racial or ethnic minority groups in Australia.	0.02	[-0.14, 0.14]	0.88	[0.73, 1.00]
10.Racial or ethnic minority groups take away jobs from other Australians.	0.65	[0.44, 0.81]	-0.07	[-0.27, 0.07]
11.The Australian way of life is weakened by people from minority racial or ethnic backgrounds maintaining their cultural beliefs and values.	0.65	[0.46, 0.83]	0.04	[-0.11, 0.13]
12.People from racial and ethnic minority groups should behave more like mainstream Australians.	0.81	[0.63, 0.95]	0.01	[-0.18, 0.13]

Deleted items highlighted in bold.

After deletion of these four items and EFA re-analysis, the two-factor solution achieved simple structure. This time, however, Item 5 did not achieve a substantial factor loading (λ_2_ = 0.390; 95% CI [0.180, 0.590]) (S1 Table); that is, the factors explained only 19% of the variance of item responses (“communality”), while 81% of the variance was explained by other sources (“uniqueness”), such as measurement error. For this reason, Item 5 was also excluded from the analysis.

### Confirmation of the factorial structure in an independent sample

The 2-factor model was then selected and its fit, examined (χ^2^(19) = 341.070, p<0.001, CFI = 0.974, RMSEA = 0.083; 90% CI [0.076, 0.091]). Since the null hypothesis of *exact-fit* was rejected (χ^2^(19) = 341.070, p<0.001), we proceeded with indices of *approximate-fit*. The CFI indicated a good fit to the data (>.960), while the RMSEA was adequate (0.5 < RMSEA ≤ 1.0). Residual correlations are displayed in S2 Table. Considering the overall good fit of the model and that all items exhibited substantial factor loadings (Table 3), the two-factor model with 8 items was accepted. “Anglo-centric/Assimilationist attitudes” (e.g. “Racial or ethnic minority groups take away jobs from other Australians”) was regarded as the first subscale, whereas the second comprised six items assessing agreement with “Inclusive/Pluralistic attitudes”

**Table 3:** Confirmatory Factor Analysis: Factor loadings (λs) and factor correlations (n = 2,443).

Item	Estimate (SE)	*p*-value	95% C.I.	CITC	LI_IRF_
**Subscale 1: Anglo-centric/Assimilationist attitudes**					
1. We need to stop people spreading dangerous ideas and stick to the way things have always been done in Australia.	0.629(0.014)	<0.001	[0.601, 0.656]	0.43	0.00
10. Racial or ethnic minority groups take away jobs from other Australians.	0.784(0.010)	<0.001	[0.764, 0.804]	0.50	0.72
11.The Australian way of life is weakened by people from minority racial or ethnic backgrounds maintaining their cultural beliefs and values.	0.856(0.009)	<0.001	[0.838, 0.874]	0.58	0.44
12.People from racial and ethnic minority groups should behave more like mainstream Australians.	0.814(0.010)	<0.001	[0.794, 0.834]	0.57	0.01
**Subscale 2: Inclusive/Pluralistic attitudes**					
4. We should do what we can to create equal conditions for different racial or ethnic groups.	0.652(0.016)	<0.001	[0.620, 0.684]	0.41	-1.58
7. People from racial or ethnic minority groups benefit Australian society.	0.627(0.016)	<0.001	[0.595, 0.658]	0.39	-1.16
8. People from racial and ethnic minority groups experience discrimination in Australia.	0.680(0.013)	<0.001	[0.655, 0.706]	0.43	-0.80
9. Something more should be done to reduce discrimination experienced by people from racial or ethnic minority groups in Australia.	0.835(0.012)	<0.001	[0.813, 0.858]	0.54	-0.86
Factor correlation (anglo-centric/assimilationist attitudes x inclusive/pluralistic attitudes)	-0.638(0.016)	<0.001	[-0.669, -0.608]	-	-

CITC = Corrected Item-Total Correlations. LI_IRF_ = Location Index based on the Item Response Function. Standardized factor loadings are displayed. Point estimates and SE were pooled across 20 imputed datasets according to Rubin’s rules. LI_IRF_ was calculated based on pooled item thresholds and factor loadings.

### Analysis of measurement invariance

Next, measurement invariance by sex, education and age was evaluated (Table 4). Regarding sex, the LRT indicated that the metric model was not statistically different from the configural model (Δ χ2 (6) = 11.86; p = 0.065), and that the scalar model was not statistically different from the metric model (Δ χ2 (16) = 24.26; p = 0.083). In other words, factor loadings and thresholds were invariant across men and women. Regarding education, although the configural model and scalar model were statistically different (Δ χ2 (6) = 19.14; p = 0.004), the fit of the (constrained) metric model improved (ΔCFI = 0.002) providing evidence of metric invariance between those with and without higher education. The same happened when metric invariance was evaluated by age; although the configural model and scalar model were statistically different (Δ χ2 (6) = 15.15; p = 0.019), the fit of the metric model (ΔCFI = 0.005) was better. When scalar invariance was evaluated, the pooled Δ χ2 was negative for both education- and age-based groups. Although a negative Δ χ2 is not interpretable (and, therefore, values were set to zero), these negative values can occur when the difference between models are small [76]. For this reason, the threshold constraints were regarded as tenable [77] and provided indirect support for scalar invariance.

**Table 4:** Measurement invariance according to sex and education.

Model	χ^2^	*df*	*p*-value	RMSEA	90% CI	CFI	Δ χ2 (df)	*p*-value	Δ CFI
**Sex**									
***Configural***	381.703	38	<0.001	0.086	[0.078, 0.094]	0.973	-	-	-
***Metric***	340.310	44	<0.001	0.074	[0.067, 0.082]	0.976	11.86 (6)	0.065	0.003
***Scalar***	428.058	60	<0.001	0.074	[0.065, 0.077]	0.971	24.26 (16)	0.083	0.005
**Education**									
***Configural***	363.867	38	<0.001	0.084	[0.076, 0.092]	0.974	**-**	**-**	**-**
***Metric***	339.008	44	<0.001	0.074	[0.067, 0.082]	0.976	19.14 (6)	0.004	0.002
***Scalar***	422.999	60	<0.001	0.070	[0.064, 0.077]	0.971	0 (6)*	1.000	-0.005
**Age**									
***Configural***	385.254	38	<0.001	0.087	[0.079, 0.094]	0.973	**-**	**-**	**-**
***Metric***	332.751	44	<0.001	0.073	[0.066, 0.081]	0.978	15.15 (6)	0.019	0.005
***Scalar***	386.834	60	<0.001	0.067	[0.061, 0.073]	0.975	0 (6)*	1.000	-0.003

χ2 = chi-square; df = degrees of freedom; RMSEA = root mean square error of approximation; CFI = comparative fit index; Δχ2 (df) = chi-square difference and degrees of freedom; ΔCFI = CFI difference.

* Negative pooled test statistic was set to zero.

### Reliability

The first subscale “Anglo-centric/Assimilationist attitudes” (Ω_H_ = 0.83, α_ORDINAL_ = 0.85, α = 0.85; 95% CI [0.84, 0.86]) showed good reliability, while the “Inclusive/Pluralistic attitudes” subscale (Ω_H_ = 0.77, α_ORDINAL_ = 0.79, α = 0.72; 95% CI [0.70, 0.73]) exhibited adequate reliability.

### Item reduction analysis

Inter-item correlations ranged from 0.29 to 0.56 (Supplementary 3) and no correlations were lower than 0.20. The CITCs ranged from 0.39 to 0.58. Within the “Anglo-centric/Assimilationist attitudes” subscale, the easiest item was “We need to stop people spreading dangerous ideas and stick to the way things have always been done in Australia” (LI_IRF_ = 0.00), while the hardest item was “Racial or ethnic minority groups take away jobs from other Australians” (LI_IRF_ = 0.72) (Table 3). That is, with respect to Item 10, respondents needed to have 0.72 standard deviations more Anglo-centric/assimilationist attitudes than the average Australian to produce an expected score of 2 out of 4. Item 10 was the *hardest* item in the “Anglo-centric/Assimilationist attitudes” subscale since its endorsement required more Anglo-centric/assimilationist attitudes than the other items. Within the “Inclusive/Pluralistic attitudes” subscale, the easiest item was “We should do what we can to create equal conditions for different racial or ethnic groups” (LI_IRF_ = -1.58), while the hardest item was “People from racial and ethnic minority groups experience discrimination in Australia.” (LI_IRF_ = -0.80). The hierarchy of item difficulties was identical when average item thresholds ($\overset{-}{\tau }$) were inspected (S4 Table).

### Construct validity

**Fig 2:**
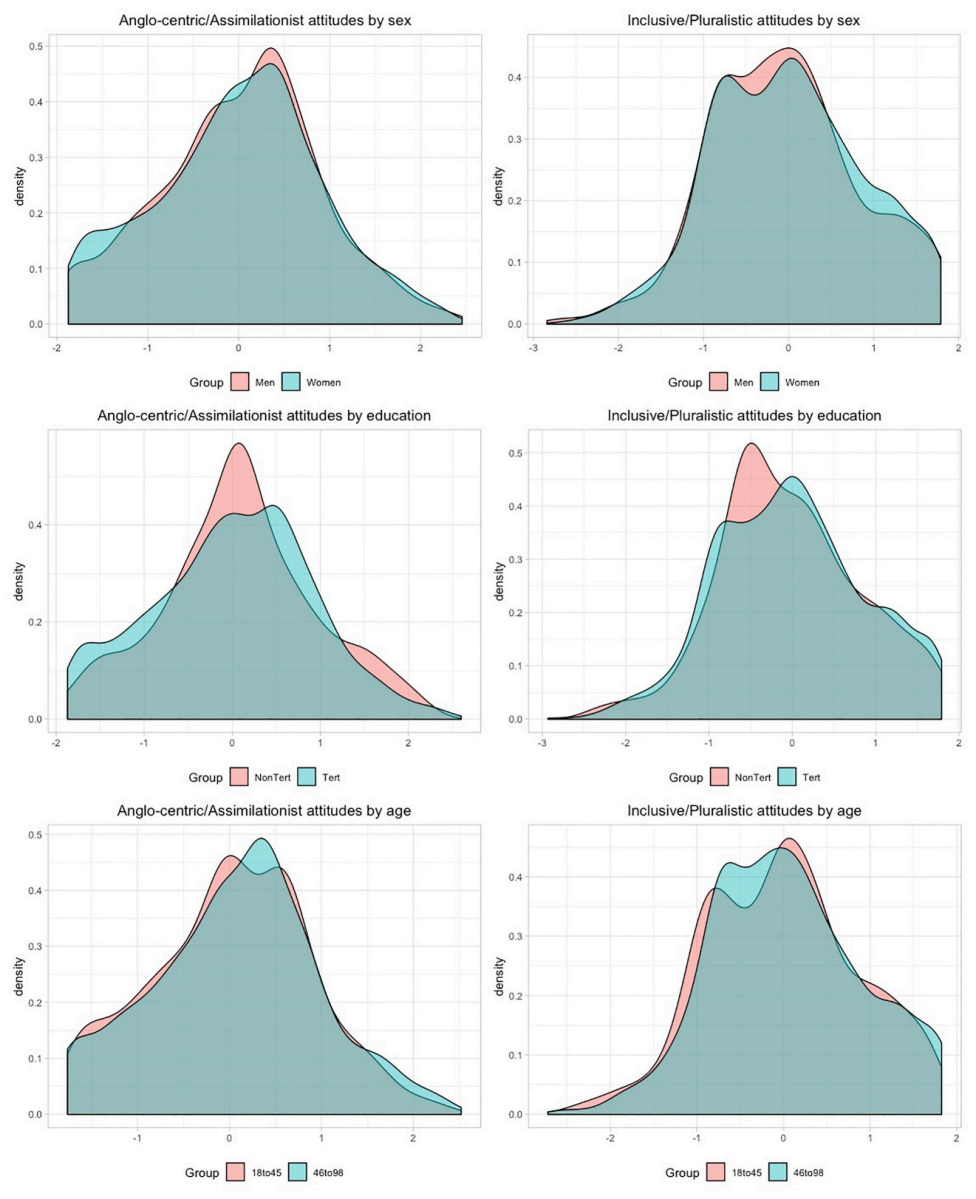
Factor scores Kernel density plots of assimilationist and pluralistic attitudes. The Kernel density plots indicate the distribution of factor scores.

Examination of criterion-related validity indicated that men (M = 0.105; 95% CI [0.014, 0.197]), participants without tertiary education (M = 0.585; 95% CI [0.474, 0.696]) and those aged 45 years and over (M = 0.373; 95% CI [0.275, 0.470]) had higher Anglo-centric/assimilationist attitudes. Furthermore, men (M = -0.116; 95% CI [-0.213, -0.020]) and participants without tertiary education (M = -0.304; 95% CI [-0.420, -0.188]) also presented lower inclusive/pluralistic attitudes. The difference in inclusive/pluralistic attitudes between participants aged 45 years and over (M = -0.045; 95% CI [-0.148, 0.057]) and their peers was close to zero. The distribution of factor scores is displayed in Fig 2.

## Discussion

The current study aimed to present the RRAMS as a measure of attitudes towards multiculturalism in Australia and to examine some of its psychometric properties using data from a nationwide sample. Results showed that the two subscales of “Anglocentric/Assimilationist attitudes” and “Inclusive/Pluralistic attitudes” are initially valid and reliable for the Australian population. In the initial stage of psychometric assessment, we identified poorly performing items, and these were excluded. One of these was Item 2 (“Some of the best people in our country are those who are challenging our government and ignoring the ‘normal’ way things are supposed to be done”), an item originally designed to reflect RWA in relation to multiculturalism. Despite its original purpose, Item 2 might not reflect the cultural and race-related topic in question. This is one possible explanation why the responses to this item were not strongly influenced by respondents’ Inclusive/Pluralistic attitudes towards multiculturalism (only 12% of the variance was explained by the supposedly corresponding factor). For instance, the wording “challenging our government” can be interpreted as referring to a general debate not necessarily reflecting ethnic-racial differences on political representation and resources distribution. Future studies might test the item fit by emphasizing ‘challenging our government’ as pressuring for a political agenda that prioritizes reducing social inequalities among ethnic-racial groups and promotion of a pluralistic society. Items 3 (“It is okay if some racial or ethnic groups have better opportunities in life than others”) and 6 (“We shouldn’t talk about racial or ethnic differences”) also performed poorly and failed to capture assimilationist views. Item 3 was designed to reflect respondent’s SDO. It was hypothesized that participants with high SDO, and thus assimilationist views of multiculturalism, would endorse the item. Contrarily to expected, these respondents might have interpreted the phrasing ‘some racial or ethnic groups’ as a reference to ethnic-racial minorities. Conservatives might perceive affirmative action and social assistance policies as privileges and can endorse the notion that minorities ‘have it easy.’ Conservative attitudes such as that of RWA and SDO have been linked to social and economic conservatism, reflecting ideologies of competition and meritocracy [78]. The ambiguity left by the item wording can thus explain its failure in discriminating assimilationist attitudes. Item 6, in turn, might have not worked in its subscale because, again contrarily to our hypothesis, respondents with high assimilationist views might be *willing* to discuss racial and ethnic differences with the intent of promoting assimilationist and racist views [79]. Therefore, the item performed poorly as respondents in the different strata of assimilationist attitudes could be prone do endorse the item for different reasons. The last deleted item was Item 5 (“Australians from an Anglo background [that is, of British descent] enjoy an advantaged position in our society”). One possible explanation for the item’s poor performance is that the recognition of privilege *does not necessarily* informs on inclusive/pluralistic attitudes. For example, a previous study in the Australian states of Queensland and New South Wales showed these as two independent dimensions [9]. The poor loading on the inclusive attitudes subscale suggests that respondents might not link acknowledgment of white privilege to notions of a pluralistic society. Taken together, these results potentially indicate that debates over multiculturalism in Australia need to promote awareness of the connection between Anglo-privilege and racism. Scholars advocate that challenging racism and privilege is as a necessary step towards promoting the abandonment of assimilationist views in favour of more inclusive perspectives [9, 13].

The subscales “Anglo-centric/Assimilationist attitudes” and “Inclusive/Pluralistic attitudes” achieved *metric invariance* and *scalar invariance* according to sex. Furthermore, the two subscales achieved *metric invariance* according education and the results also (indirectly) supported *scalar invariance*. That is, “Anglo-centric/Assimilationist attitudes” and “Inclusive/Pluralistic attitudes” influenced the item responses the same way in each group (*metric invariance*) and the items were not more difficult for one group compared to another (*scalar invariance*). The RRAMS items can thus be used to compare men/women, participants with/without tertiary education and young/older participants, and the scores will reflect *true differences* regarding “Anglo-centric/Assimilationist attitudes” and “Inclusive/Pluralistic attitudes” rather than measurement bias [35].

After ensuring measurement invariance between subgroups, we compared the factor scores between men and women, participants with and without tertiary education, and participants up to and over 45 years of age. The stronger predictor of assimilationist *and* inclusive attitudes was education, while sex also influenced both constructs. Furthermore, older individuals were more likely to have higher assimilationist attitudes. The role of education in promoting inclusive/pluralistic has been previously established [22, 70] and suggests education as an important target for future interventions aimed at promoting multiculturalism in Australia. The results also indicated that men and older individuals had stronger assimilationist attitudes in comparison with women and younger counterparts [71]. In general, the associations of the two subscales with sex, education, and age conformed to the theoretical expectations and provide further evidence of the RRAMS’ construct validity.

With regards to reliability, the “Anglo-centric/Assimilationist attitudes” and “Inclusive/Pluralistic attitudes” subscales showed adequate reliability (>.70) [80], since values between .70 and .80 are considered appropriate for research purposes [81]. In case the RRAMS is used in the future in high-stakes scenarios (i.e. where decisions need to be made based on scale scores) [82], new items should be developed to increase reliability.

In the item reduction analysis, all items displayed moderate inter-item correlations and CITC, so no items needed to be removed. The item with the smallest CITC was Item 7 (“People from racial or ethnic minority groups benefit Australian society”), followed by Item 4 (“We should do what we can to create equal conditions for different racial or ethnic groups.”). Since reliability was only modest, we considered that further shortening the scale would be more detrimental in terms of reliability and content validity than beneficial as a means of creating a briefer measure. In addition, with the exception of Item 1 (“We need to stop people spreading dangerous ideas and stick to the way things have always been done in Australia.”) and Item 12 (“People from racial and ethnic minority groups should behave more like mainstream Australians.”), items difficulties were spread across the latent trait. Once again, although Item 1 or Item 2 could potentially be removed due to similar difficulties, we believe removing additional items would be detrimental to content validity and the psychometric properties of the scale.

One limitation of the current study was that we were not able to evaluate convergent and discriminant validity. The RRAMS was originally applied at the 2013 NDTIS, a study that focused on collecting information on the use of dental services in Australia and did not include other psychosocial measures. For this reason, we considered known-groups validity to be the best strategy to investigate the RRAMS’ construct validity. While the results from known-groups validity were in accordance with theoretical expectations (e.g. inclusive attitudes were more present in individuals with more education), future studies need also to investigate other forms of validity, such as convergent/discriminant and predictive validity. For example, future studies should evaluate whether the scores from the “Inclusive/Pluralistic attitudes” subscale are positively correlated (i.e. convergent validity) with scores from other instruments evaluating multiculturalist and inclusive attitudes. Our analyses did not account for sampling weights, meaning that our sample is not representative of the Australian population. It is important to highlight, however, that our study included Australians from all age groups and socioeconomic backgrounds across all states and territories of the country. Furthermore, to the best of our knowledge, this is the largest sample in which a measure of attitudes towards multiculturalism has been employed in Australia. Lack of representativeness and its implications to the validity of scientific findings are central to longstanding discussions in the literature [83]. Because the purpose of the current analysis was to assess the psychometric properties of the RRAMS, as opposed to purely describe prevalence estimates, we do not believe that the lack of representativeness of our sample limits the validity of inferences made here. The fact that a study sample is representative of some larger population does not mean that the associations between variables in the sample will apply to every subgroup of the population. The overall association is simply an average value that has been balanced according to the distribution of people in these subgroups. If a sample that is representative of the sex distribution in the target population, the results will not necessarily be apply to both males and females, but only to a hypothetical participant that is “weighted” on sex. Subgroups analyses are necessary if one wishes to investigate relationships between variables by subgroups, which we have performed during the criterion validity assessment stage.

In conclusion, we successfully developed a comprehensive race-related attitudes and multiculturalism scale to the Australian context. We used robust, cutting edge psychometric techniques and data from a large, nation-wide survey. The small number of items (eight) means the instrument will likely be readily used by policy makers and in ensuing research. Future studies should assess the scaling properties of the instrument by using parametric and non-parametric Item Response Theory techniques. The instrument may, nevertheless, be useful to inform on multiculturalism attitudes across the country and hopefully contribute to a public debate aimed to promote multiculturalist inclusive attitudes with the potential to increase social cohesion in Australia.

## Supporting information

S1 File (R)

S1 Table Exploratory Factor Analysis: Factor loadings (λs) and bootstrapped 95% CI. (DOCX)

S2 Table Matrix of residual correlations. (DOCX)

S3 Table Matrix of inter-item correlations. (DOCX)

S4 Table Confirmatory Factor Analysis: Item thresholds (τ), item locations (β) and item difficulties (LIIRF). (DOCX)

## References

[1] Berman G, Paradies Y. Racism, disadvantage and multiculturalism: towards effective anti-racist praxis. Ethnic and Racial Studies. 2010;33(2):214–32.

[2] Elias A, Paradies Y. Estimating the mental health costs of racial discrimination. BMC Public Health. 2016;16(1):1205 10.1186/s12889-016-3868-1 27899096 PMC5129635

[3] Hage G. Analysing multiculturalism today In: Bennett T, Frow J, editors. The SAGE handbook of cultural analysis: SAGE Publishing; 2007 p. 488–509.

[4] Habtegiorgis AE, Paradies YC, Dunn KM. Are racist attitudes related to experiences of racial discrimination? Within sample testing utilising nationally representative survey data. Soc Sci Res. 2014;47:178–91. 10.1016/j.ssresearch.2014.05.002 24913953

[5] Kauff M, Wagner U. Valuable Therefore Not Threatening The Influence of Diversity Beliefs on Discrimination Against Immigrants2012. 714–21 p.

[6] Modood T. Multiculturalism In: Ritzer G, editor. The Blackwell Encyclopedia of Sociology: John Wiley & Sons; 2016.

[7] Grigg K, Manderson L. The Australian Racism, Acceptance, and Cultural-Ethnocentrism Scale (RACES): item response theory findings. International Journal for Equity in Health. 2016;15(1):49. 26987795 10.1186/s12939-016-0338-4 PMC4794855

[8] Hage GaB. Analysing Multiculturalism Today The SAGE Handbook of Cultural Analysis 2008 p. 488–509.

[9] Forrest J, Dunn K. ‘Core’Culture Hegemony and Multiculturalism. Ethnicities. 2016;6(2):203–30.

[10] Vala J, Costa-Lopes R. Intergroup relations In: Smelser NJ, Baltes PB, editors. International Encyclopedia of the Behavioral Sciences. 2nd edition ed: Elsevier; 2015.

[11] Verkuyten M. Support for Multiculturalism and Minority Rights: The Role of National Identification and Out-group Threat. Social Justice Research. 2009;22(1):31–52.

[12] Morrison KR, Ybarra O. The effects of realistic threat and group identification on social dominance orientation. J Exp Soc Psychol. 2008;44(1):156–63.

[13] Dunn K, Nelson JK. Challenging the Public Denial of Racism for a Deeper Multiculturalism. Journal of Intercultural Studies. 2011;32(6):587–602.

[14] Pratto F, Sidanius J, Levin S. Social dominance theory and the dynamics of intergroup relations: Taking stock and looking forward. European Review of Social Psychology. 2006;17(1):271–320.

[15] Adorno TW, Frenkel-Brunswik E, Levinson D, Sandford RN. The Authoritarian personality. New York: Harper; 1950.

[16] Bizumic B, Duckitt J. Investigating right wing authoritarianism with a very short authoritarianism scale. Journal of Social and Political Psychology. 2018;6(1):129–50.

[17] Duckitt J. Authoritarianism and dogmatism In: Leary M, Hoyle R, editors. Handbook of individual differences in social behavior. New York: Guilford Press; 2009 p. 298–317.

[18] Perry R, Sibley CG. Big-Five personality prospectively predicts Social Dominance Orientation and Right-Wing Authoritarianism. Pers Individ Dif. 2012;52(1):3–8.

[19] Sumino T. National identity and public attitudes toward multiculturalism in Canada: Testing the indirect effect via perceived collective threat. Canadian Journal of Behavioural Science / Revue canadienne des sciences du comportement. 2017;49(3):183–94.

[20] Doane A. Beyond Color-blindness: (Re) Theorizing Racial Ideology. Sociological Perspectives. 2017;60(5):975–91.

[21] Bonilla-Silva E. Racism without racists: Color-Blind Racism and the persistence of racial inequality in the United States. New York: Rowman & Littlefield; 2003.

[22] Dunn K, Blair K, Alam O, Kamp A. Australians’ Views on Cultural Diversity, Nation and Migration, 2015–16. Cosmopolitan Civil Societies: An Interdisciplinary Journal. 2017;9(3):61–84.

[23] Marwick A, Ansari Z, Sullivan M, Parsons L, McNeil J. Inequalities in the social determinants of health of Aboriginal and Torres Strait Islander People—A cross-sectional population-based study in the Australian state of Victoria. International Journal for Equity in Health. 2014;13(91):1–12. 25326177 10.1186/s12939-014-0091-5 PMC4209035

[24] Curthoys A. An uneasy conversation: the multicultural and the Indigenous In: Docker J, Fischer G, editors. Race, colour and identity in Australia and New Zealand. Sydney: UNSW Press; 2000 p. 21–36.

[25] Denson N, Ovenden G, Wright L, Paradies Y, Priest N. The development and validation of intercultural understanding (ICU) instruments for teachers and students in primary and secondary schools. Intercultural Education. 2017;28(3):231–49.

[26] Australian Institute of Health and Welfare. Australia’s welfare 2017. Canberra: Australian Institute of Health and Welfare, 2017.

[27] R Core Team. R: A language and environment for statistical computing. 2013.

[28] Rosseel Y. Lavaan: An R package for structural equation modeling and more. Version 0.5–12 (BETA). Journal of statistical software. 2012;48(2):1–36.

[29] Jorgensen T, Pornprasertmanit S, Schoemann A, Rosseel Y. semTools: Useful tools for structural equation modeling. R Package Version 0.5–1. 2018.

[30] Haynes SN, Richard DCS, Kubany ES. Content validity in psychological assessment: A functional approach to concepts and methods. Psychol Assess. 1995;7(3):238–47.

[31] Blair K, Dunn, K., Kamp, A., & Alam, O. Challenging Racism Project 2015–16 National Survey Report. In: University. SWS, editor. 2017.

[32] VicHealth. Findings from the 2013 survey of Victorians’ attitudes to race and cultural diversity. In: Foundation VHP, editor. Melbourne, Australia2014.

[33] Rees S, Silove D, Chey T, Ivancic L, Steel Z, Creamer M, et al Lifetime prevalence of gender-based violence in women and the relationship with mental disorders and psychosocial function. Journal of the American Medical Association. 2011;306(5):513–21. 10.1001/jama.2011.1098 21813429

[34] Fokkema M, Greiff S. How Performing PCA and CFA on the Same Data Equals Trouble. Hogrefe Publishing; 2017.

[35] Kline RB. Principles and practice of structural equation modeling. New York, NY: Guilford publications; 2015.

[36] Fabrigar LR, Wegener DT. Exploratory factor analysis: Oxford University Press; 2011.

[37] Cattell RB. The scree test for the number of factors. Multivariate behavioral research. 1966;1(2):245–76. 10.1207/s15327906mbr0102_10 26828106

[38] Horn JL. A rationale and test for the number of factors in factor analysis. Psychometrika. 1965;30(2):179–85. 14306381 10.1007/BF02289447

[39] Lawley DN. Vi.—the estimation of factor loadings by the method of maximum likelihood. Proceedings of the Royal Society of Edinburgh. 1940;60(1):64–82.

[40] Harman HH. Modern factor analysis: University of Chicago press; 1976.

[41] Efron B, Tibshirani RJ. An introduction to the bootstrap: CRC press; 1994.

[42] Muthén B. A general structural equation model with dichotomous, ordered categorical, and continuous latent variable indicators. Psychometrika. 1984;49(1):115–32.

[43] Asparouhov T, Muthén B. Simple second order chi-square correction. Mplus technical appendix. 2010.

[44] Van Buuren S. Multiple imputation of discrete and continuous data by fully conditional specification. Stat Methods Med Res. 2007;16(3):219–42. 10.1177/0962280206074463 17621469

[45] Rubin DB. Multiple imputation for nonresponse in surveys: John Wiley & Sons; 2004.

[46] Yu C-Y. Evaluating cutoff criteria of model fit indices for latent variable models with binary and continuous outcomes: University of California, Los Angeles Los Angeles; 2002.

[47] Van Prooijen J-W, Van Der Kloot WA. Confirmatory analysis of exploratively obtained factor structures. Educ Psychol Meas. 2001;61(5):777–92.

[48] Saris WE, Satorra A, Van der Veld WM. Testing structural equation models or detection of misspecifications? Structural Equation Modeling. 2009;16(4):561–82.

[49] Wu H, Estabrook R. Identification of confirmatory factor analysis models of different levels of invariance for ordered categorical outcomes. Psychometrika. 2016;81(4):1014–45. 10.1007/s11336-016-9506-0 27402166 PMC5458787

[50] Satorra A. Scaled and adjusted restricted tests in multi-sample analysis of moment structures Innovations in multivariate statistical analysis: Springer; 2000 p. 233–47.

[51] Li K-H, Meng X-L, Raghunathan TE, Rubin DB. Significance levels from repeated p-values with multiply-imputed data. Statistica Sinica. 1991:65–92.

[52] Cheung GW, Rensvold RB. Evaluating goodness-of-fit indexes for testing measurement invariance. Structural equation modeling. 2002;9(2):233–55.

[53] Meade AW, Johnson EC, Braddy PW. Power and sensitivity of alternative fit indices in tests of measurement invariance. J Appl Psychol. 2008;93(3):568 10.1037/0021-9010.93.3.568 18457487

[54] Byrne BM, Shavelson RJ, Muthén B. Testing for the equivalence of factor covariance and mean structures: The issue of partial measurement invariance. Psychol Bull. 1989;105(3):456.

[55] McDonald RP. Test theory: A unified treatment: Psychology Press; 2013.

[56] Zumbo BD, Gadermann AM, Zeisser C. Ordinal versions of coefficients alpha and theta for Likert rating scales. Journal of modern applied statistical methods. 2007;6(1):4.

[57] Dunn TJ, Baguley T, Brunsden V. From alpha to omega: A practical solution to the pervasive problem of internal consistency estimation. Br J Psychol. 2014;105(3):399–412. 10.1111/bjop.12046 24844115

[58] Turner HJ, Natesan P, Henson RK. Performance Evaluation of Confidence Intervals for Ordinal Coefficient Alpha. Journal of Modern Applied Statistical Methods. 2017;16(2):9.

[59] Piedmont RL. Inter-item correlations. Encyclopedia of quality of life and well-being research. 2014:3303–4.

[60] Rolstad S, Adler J, Rydén A. Response burden and questionnaire length: is shorter better? A review and meta-analysis. Value Health. 2011;14(8):1101–8. 10.1016/j.jval.2011.06.003 22152180

[61] Zijlmans EAO, Tijmstra J, der Ark V, Andries L, Sijtsma K. Item-score reliability as a selection tool in test construction. Front Psychol. 2018;9:2298 10.3389/fpsyg.2018.02298 30687144 PMC6336834

[62] Guilford JP. The correlation of an item with a composite of the remaining items in a test. Educ Psychol Meas. 1953;13(1):87–93.

[63] Gardner PL. Measuring attitudes to science: Unidimensionality and internal consistency revisited. Research in science education. 1995;25(3):283–9.

[64] Kendall SM. Rank correlation. Van Nostrand’s Scientific Encyclopedia. 1948.

[65] Embretson SE. The new rules of measurement. Psychol Assess. 1996;8(4):341.

[66] Ali US, Chang HH, Anderson CJ. Location Indices for Ordinal Polytomous Items Based on Item Response Theory. ETS Research Report Series. 2015;2015(2):1–13.

[67] Muthen BO. Instructional Sensitivity in Mathematics Achievement Test Items: Application of a New IRT-Based Detection Technique. 1988.

[68] Ferrando PJ. Difficulty, discrimination, and information indices in the linear factor analysis model for continuous item responses. Appl Psychol Meas. 2009;33(1):9–24.

[69] Davidson M. Known-groups validity Encyclopedia of quality of life and well-being research: Springer; 2014 p. 3481–2.

[70] Dandy J, Pe-Pua R. Attitudes to multiculturalism, immigration and cultural diversity: Comparison of dominant and non-dominant groups in three Australian states. International Journal of Intercultural Relations. 2010;34(1):34–46.

[71] Dunn KM, Forrest J, Burnley I, McDonald A. Constructing Racism in Australia. Australian Journal of Social Issues. 2004;39(4):409–30.

[72] Cohen J. Statistical power analysis for the behavioral sciences. 2nd Hillsdale, NJ: erlbaum; 1988.

[73] Hancock GR. Effect size, power, and sample size determination for structured means modeling and MIMIC approaches to between-groups hypothesis testing of means on a single latent construct. Psychometrika. 2001;66(3):373–88.

[74] Rabe-Hesketh S, Skrondal A. Assigning values to latent variables Generalized latent variable modeling: Multilevel, longitudinal, and structural equation models: Chapman and Hall/CRC; 2004.

[75] Sheather SJ, Jones MC. A reliable data-based bandwidth selection method for kernel density estimation. Journal of the Royal Statistical Society Series B (Methodological). 1991:683–90.

[76] Satorra A, Bentler PM. Ensuring positiveness of the scaled difference chi-square test statistic. Psychometrika. 2010;75(2):243–8. 10.1007/s11336-009-9135-y 20640194 PMC2905175

[77] Marshall GN, Miles JN, Stewart SH. Anxiety sensitivity and PTSD symptom severity are reciprocally related: Evidence from a longitudinal study of physical trauma survivors. J Abnorm Psychol. 2010;119(1):143 10.1037/a0018009 20141251 PMC2820125

[78] Harnish RJ, Bridges KR, Gump JT. Predicting Economic, Social, and Foreign Policy Conservatism: the Role of Right-Wing Authoritarianism, Social Dominance Orientation, Moral Foundations Orientation, and Religious Fundamentalism. Current Psychology. 2017;37(3):668–79.

[79] Faulkner N, Bliuc A-M. ‘It’s okay to be racist’: moral disengagement in online discussions of racist incidents in Australia. Ethnic Racial Stud. 2016;39(14):2545–63.

[80] Nunnally JC, Bernstein IH. Psychometric theory: McGraw-Hill New York; 1967.

[81] Furr RM, Bacharach VR. Psychometrics: an introduction. Thousand Oaks, CA: Sage; 2013.

[82] Wells CS, Wollack JA. An instructor’s guide to understanding test reliability. Testing & Evaluation Services University of Wisconsin. 2003.

[83] Bollen KA, Biemer PP, Karr AF, Tueller S, Berzofsky ME. Are Survey Weights Needed? A Review of Diagnostic Tests in Regression Analysis. Annual Review of Statistics and Its Application. 2016;3(1):375–92.

