# Supplementary material for: Development and initial psychometric assessment of the race-related attitudes and multiculturalism scale in Australia
Source: PLoS One. 2020 Apr 1;15(4):e0230724. doi: 10.1371/journal.pone.0230724 (PMC7112161; doi:10.1371/journal.pone.0230724)
Supplement: S1 Table — (DOCX) [file pone.0230724.s002.docx]

**Supplementary Table 1.** Exploratory Factor Analysis: Factor Loadings (λs) and Bootstrapped 95% CI.

|  | Factor 1 | | Factor 2 |  |
| --- | --- | --- | --- | --- |
| Item | Estimate | 95% C.I. | Estimate | 95% C.I. |
| 1. We need to stop people spreading dangerous ideas and stick to the way things have always been done in Australia. | 0.60 | [0.43, 0.74] | -0.09 | [-0.23, 0.04] |
| 4. We should do what we can to create equal conditions for different racial or ethnic groups. | -0.12 | [-0.26, 0.00] | 0.57 | [0.41, 0.72] |
| 5. Australians from an Anglo background (that is, of British descent) enjoy an advantaged position in our society. | **-0.06** | **[-0.26, 0.09]** | **0.39** | **[0.18, 0.59]** |
| 7. People from racial or ethnic minority groups benefit Australian society. | -0.06 | [-0.24, 0.10] | 0.45 | [0.23, 0.67] |
| 8. People from racial and ethnic minority groups experience discrimination in Australia. | -0.02 | [-0.17, 0.09] | 0.71 | [0.54, 0.86] |
| 9. Something more should be done to reduce discrimination experienced by people from racial or ethnic minority groups in Australia. | 0.04 | [-0.09, 0.15] | 0.91 | [0.79, 1.00] |
| 10.Racial or ethnic minority groups take away jobs from other Australians. | 0.62 | [0.40, 0.79] | -0.09 | [-0.30, 0.07] |
| 11.The Australian way of life is weakened by people from minority racial or ethnic backgrounds maintaining their cultural beliefs and values. | 0.83 | [0.71, 0.93] | 0.04 | [-0.08, 0.13] |
| 12.People from racial and ethnic minority groups should behave more like mainstream Australians. | 0.82 | [0.68, 0.94] | 0.02 | [-0.12, 0.13] |

Note. Deleted items were highlighted in bold. This is the result of the Exploratory Factor Analysis after items 2, 3 and 6 were deleted.
