# Supplementary material for: Development and initial psychometric assessment of the race-related attitudes and multiculturalism scale in Australia
Source: PLoS One. 2020 Apr 1;15(4):e0230724. doi: 10.1371/journal.pone.0230724 (PMC7112161; doi:10.1371/journal.pone.0230724)
Supplement: S2 Table — (DOCX) [file pone.0230724.s003.docx]

**Supplementary Table 2.** Matrix of residual correlations.

|  | Item 1 | Item 10 | Item 11 | Item 12 | Item 4 | Item 7 | Item 8 | Item 9 |
| --- | --- | --- | --- | --- | --- | --- | --- | --- |
| Item 1 | 0.000 |  |  |  |  |  |  |  |
| Item 10 | -0.032 | 0.000 |  |  |  |  |  |  |
| Item 11 | -0.019 | -0.003 | 0.000 |  |  |  |  |  |
| Item 12 | 0.051 | -0.040 | 0.018 | 0.000 |  |  |  |  |
| Item 4 | 0.033 | -0.061 | -0.006 | 0.045 | 0.000 |  |  |  |
| Item 7 | -0.034 | -0.088 | -0.049 | -0.008 | 0.020 | 0.000 |  |  |
| Item 8 | -0.022 | 0.008 | 0.060 | 0.020 | -0.053 | -0.055 | 0.000 |  |
| Item 9 | 0.061 | -0.036 | 0.037 | 0.043 | 0.011 | -0.059 | 0.055 | 0.000 |

Note. The residual correlations matrix displays the observed correlation between item responses after the influence of the latent factors was accounted by the model.
