# Supplementary material for: Development and initial psychometric assessment of the race-related attitudes and multiculturalism scale in Australia
Source: PLoS One. 2020 Apr 1;15(4):e0230724. doi: 10.1371/journal.pone.0230724 (PMC7112161; doi:10.1371/journal.pone.0230724)
Supplement: S3 Table — (DOCX) [file pone.0230724.s004.docx]

**Supplementary Table 3.** Matrix of inter-item correlations.

|  | Item 1 | Item 10 | Item 11 | Item 12 | Item 4 | Item 7 | Item 8 | Item 9 |
| --- | --- | --- | --- | --- | --- | --- | --- | --- |
| Item 1 | 1.000 |  |  |  |  |  |  |  |
| Item 10 | 0.341 | 1.000 |  |  |  |  |  |  |
| Item 11 | 0.394 | 0.537 | 1.000 |  |  |  |  |  |
| Item 12 | 0.429 | 0.448 | 0.563 | 1.000 |  |  |  |  |
| Item 4 | -0.157 | -0.294 | -0.275 | -0.204 | 1.000 |  |  |  |
| Item 7 | -0.212 | -0.329 | -0.314 | -0.250 | 0.345 | 1.000 |  |  |
| Item 8 | -0.222 | -0.268 | -0.249 | -0.252 | 0.293 | 0.292 | 1.000 |  |
| Item 9 | -0.211 | -0.367 | -0.332 | -0.298 | 0.438 | 0.389 | 0.516 | 1.000 |

Note. The inter-item correlations matrix displays the observed marginal correlations between item responses.
