# Supplementary material for: Development and initial psychometric assessment of the race-related attitudes and multiculturalism scale in Australia
Source: PLoS One. 2020 Apr 1;15(4):e0230724. doi: 10.1371/journal.pone.0230724 (PMC7112161; doi:10.1371/journal.pone.0230724)
Supplement: S4 Table — (DOCX) [file pone.0230724.s005.docx]

**Supplementary Table 4.** Confirmatory Factor Analysis: Item Thresholds (τ), Item Locations (β) and Item Difficulties (LI_IRF_).

| Item | τ_1_ (SE) | τ_2_ (SE) | τ_3_ (SE) | τ_4_ (SE) | $\bar{}$ | β_1_ | β_2_ | β_3_ | β_4_ | LI_IRF_ |
| --- | --- | --- | --- | --- | --- | --- | --- | --- | --- | --- |
| Subscale 1: Anglo-centric/Assimilationist attitudes |  |  |  |  |  |  |  |  |  |  |
| 1. We need to stop people spreading dangerous ideas and stick to the way things have always been done in Australia. | -0.881  (0.030) | -0.372  (0.026) | 0.422  (0.026) | 0.834  (0.029) | 0.001 | -1.400 | -0.591 | 0.671 | 1.326 | 0.005 |
| 10. Racial or ethnic minority groups take away jobs from other Australians. | -0.455  (0.027) | 0.150  (0.026) | 1.020  (0.031) | 1.504  (0.040) | 0.555 | -0.580 | 0.191 | 1.301 | 1.918 | 0.716 |
| 11.The Australian way of life is weakened by people from minority racial or ethnic backgrounds maintaining their cultural beliefs and values. | -0.474  (0.027) | 0.055  (0.026) | 0.709  (0.028) | 1.228  (0.034) | 0.379 | -0.554 | 0.064 | 0.828 | 1.434 | 0.444 |
| 12.People from racial and ethnic minority groups should behave more like mainstream Australians. | -0.989  (0.031) | -0.417  (0.026) | 0.442  (0.027) | 0.995  (0.031) | 0.008 | -1.215 | -0.512 | 0.543 | 1.222 | 0.011 |
| Subscale 2: Inclusive/Pluralistic attitudes |  |  |  |  |  |  |  |  |  |  |
| 4. We should do what we can to create equal conditions for different racial or ethnic groups. | -1.768  (0.047) | -1.470  (0.039) | -0.753  (0.028) | -0.084  (0.026) | -1.019 | -2.712 | -2.255 | -1.155 | -0.129 | -1.579 |
| 7. People from racial or ethnic minority groups benefit Australian society. | -1.698  (0.045) | -1.283  (0.035) | -0.318  (0.026) | 0.432  (0.027) | -0.717 | -2.708 | -2.046 | -0.507 | 0.689 | -1.165 |
| 8. People from racial and ethnic minority groups experience discrimination in Australia. | -1.624  (0.043) | -1.137  (0.033) | -0.120  (0.033) | 0.811  (0.029) | -0.517 | -2.388 | -1.672 | -0.176 | 1.193 | -0.801 |
| 9. Something more should be done to reduce discrimination experienced by people from racial or ethnic minority groups in Australia. | -1.794  (0.046) | -1.294  (0.035) | -0.250  (0.026) | 0.540  (0.027) | -0.699 | -2.095 | -1.550 | -0.299 | 0.647 | -0.858 |

Note. τ*_i_* = item thresholds of adjacent categories *i* and *i+1*. β*_i_* = item locations of adjacent categories *i* and *i+1*. $\bar{}$ = average item threshold. LI_IRF_ = item location based on the item response function.
